# A Rare Complication of Dental Care: A Case of Subcutaneous Emphysema, Pneumomediastinum, and Pneumopericardium

**DOI:** 10.7759/cureus.85035

**Published:** 2025-05-29

**Authors:** Caroline Henin, Laure Watelet, Vanessa Wauters

**Affiliations:** 1 Emergency Medicine, Cliniques Universitaires Saint-Luc, Woluwe-Saint-Lambert, BEL; 2 Emergency Medicine, Centres Hospitaliers Universitaires (CHU) Helora, Nivelles, BEL

**Keywords:** air-driven dental tools, dental procedure complications, pneumomediastinum, pneumopericardium, subcutaneous emphysema

## Abstract

This article reports the rare case of a 64-year-old man who presented at the emergency department with interscapular pain following a dental procedure. Imaging revealed subcutaneous emphysema, pneumomediastinum, and pneumopericardium, resulting from the use of an air-driven tool during the treatment of a molar implant. Although uncommon, such iatrogenic complications can lead to serious consequences and should be promptly recognized and managed in emergency settings to prevent further deterioration.

## Introduction

In modern healthcare practice, routine procedures such as dental treatments are often considered safe and minimally invasive. However, any dental procedure carries a number of potential complications. The most common complications are either infectious, due to bacterial contamination of the mouth, or mechanical, resulting from the procedure itself, such as pain, swelling, or bleeding [1]. The vast majority of them are benign. However, some complications are less common and can be serious or even fatal, requiring patients to seek emergency care. Understanding the potential risks associated with each procedure and instrument is important, and patients should be informed about the symptoms that require medical attention.

This paper discusses the curious case of a 64-year-old man who presented to the emergency department with interscapular pain following a dental appointment and highlights how even a common dental appointment can lead to serious and potentially life-threatening complications.

## Case presentation

A 64-year-old man presented to the emergency department with interscapular pain. The previous day, he had a routine annual dental appointment. The dentist used a pneumatic air-driven tool to reach and clean a gingival recess around a lower left molar implant. This handpiece operates by converting compressed air into rotary motion. As soon as the procedure began, the patient experienced a sudden swelling and crepitus around the jaw angle and in the cheek. Later that day, the symptoms had migrated to the throat and neck area, with swallowing discomfort. The next morning, he woke up with thoracic pain and decided to seek emergency care. His medical history was unremarkable, and he had no prior history of respiratory or cardiovascular issues. The pain was described as a constant interscapular oppression, not exacerbated by respiration or movements. No other symptoms were depicted, e.g., no dyspnea. Upon examination, a subcutaneous crepitus could barely be felt in the neck and left cheek area. All vital signs were normal, the electrocardiogram (ECG) didn't show any changes, including no repolarization disorders and no microvoltage, and the bloodwork revealed no abnormalities.

**Figure 1:**
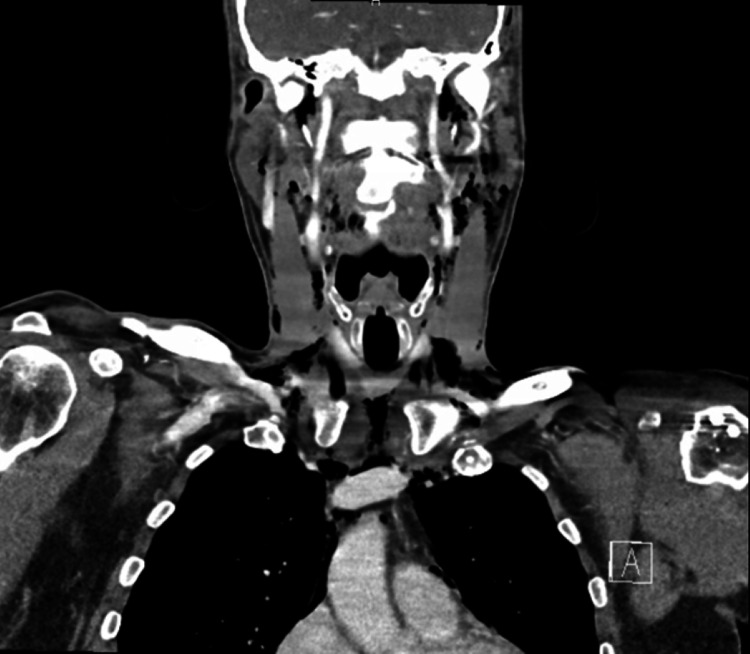
Contrast-enhanced CT scan of the neck and thoracic area showing extensive subcutaneous emphysema CT: computed tomography

**Figure 2:**
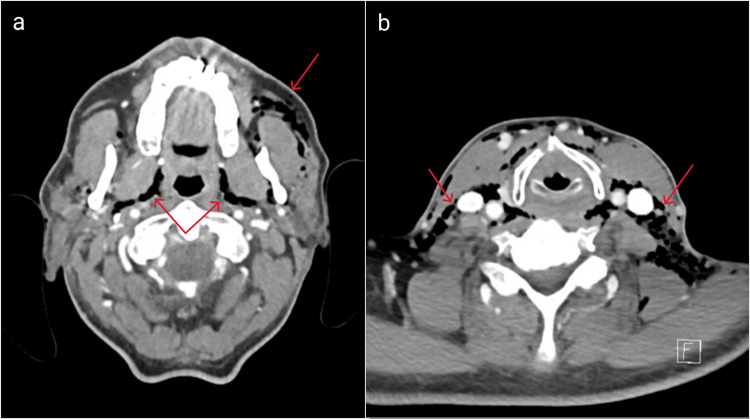
Multiple CT scan views of the neck area: (a) emphysema surrounding the lower left molar region and the floor of the mouth and (b) subcutaneous emphysema descending on the neck CT: computed tomography

**Figure 3:**
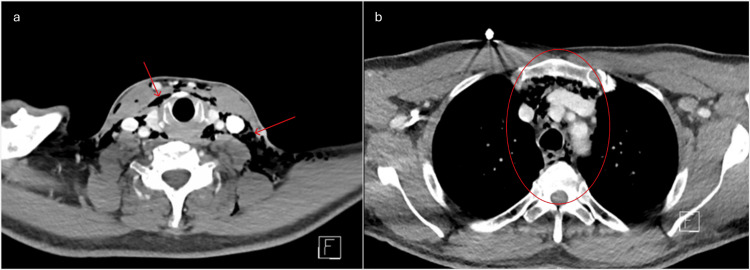
Multiple CT scan views of the thoracic area: (a) emphysema surrounding the neck vessels and the trachea and (b) emphysema extending into the mediastinum CT: computed tomography

**Figure 4:**
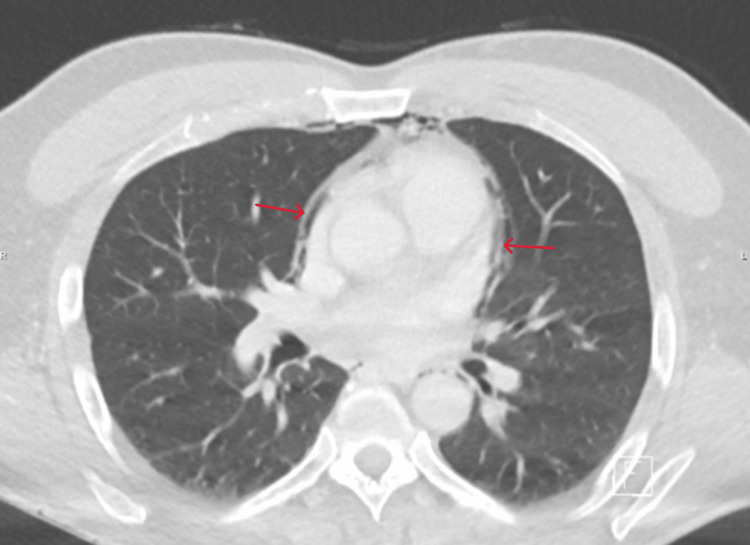
CT scan showing pneumopericardium CT: computed tomography

**Figure 5:**
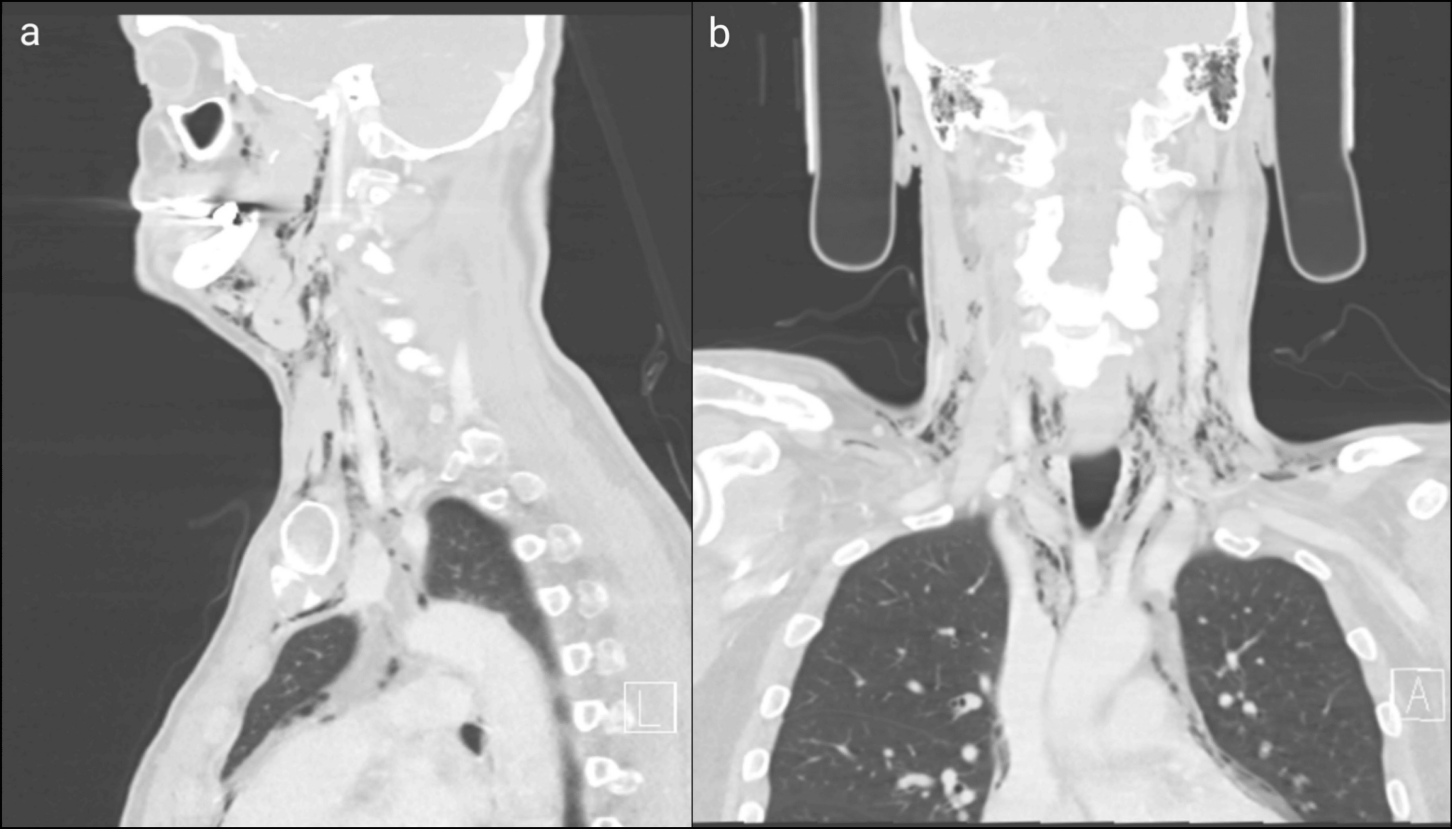
Multiple CT scan views showing extended emphysema: (a) sagittal plane and (b) coronal plane CT: computed tomography

A contrast-enhanced computed tomography (CT) scan of the neck and thoracic area was conducted (Figures 1-5).

The scan showed an extended subcutaneous emphysema, with air infiltration originating from the molar area, involving the floor of the mouth, following the neck muscles and blood vessels and the trachea and extending into the mediastinum and pericardium, most likely caused by an inadvertent air injection during the dental procedure [2,3].

After surgical advice, the patient was treated conservatively with close monitoring and prophylactic amoxicillin-clavulanic acid for four days [4]. Within a few days, all symptoms gradually resolved without further intervention. A follow-up cardiac ultrasound was performed a few days later and showed residual pneumopericardium with no evidence of complications or hemodynamic impact.

## Discussion

The aetiology of subcutaneous emphysema is diverse. Some of the major causes are either traumatic, infectious, or secondary to invasive procedures such as laparoscopic or thoracoscopic surgeries, bronchoscopy, and mechanical ventilation.

Subcutaneous emphysema is also a rare but documented complication that can occur following dental treatment, especially procedures involving high-pressure air tools, during which the increased intraoral pressure from the equipment can lead to the disruption of the dental alveolar structures, allowing air to enter the subcutaneous tissues [5-7]. Although this is usually benign, the infectious risk due to the bacterial colonization of the mouth is one of the main concerns. In this case, the air had even reached the mediastinum and the pericardium, leading to a more dangerous situation. Risk factors may include pre-existing pulmonary pathology, recent upper respiratory infections, or improper use of dental tools [8]. 

Emphysema following dental procedures is usually a self-limiting condition that resolves without the need for further interventions. Management typically involves conservative measures, including close monitoring and prophylactic antibiotics, as employed in this case, to cover the most common oral pathogens. Invasive measures, such as surgical intervention or drainage, should be reserved for severe cases or for specific situations such as pneumothorax or tamponade [9].

While the overall incidence is low, awareness of such potential complications is essential for both dental and medical professionals. Prompt recognition and appropriate management are key to preventing more serious situations, including respiratory failure or infection, such as oral or cervical abscess, or mediastinitis.

## Conclusions

This case underlines the importance of recognizing unusual but potentially serious complications following routine dental procedures. Early recognition of subcutaneous emphysema and pneumomediastinum is essential for the effective management and prevention of further complications. Dentists and emergency care providers must be vigilant and proactive when these symptoms occur to ensure adequate diagnosis, treatment, and follow-up. Furthermore, patient education and dental practice protocols could reinforce prevention.
